# Transparent Conductive Copper-Doped Zinc Oxide (ZnO:Cu) Thin Films: PVco-D Fabrication and Applications in Perovskite Solar Cells

**DOI:** 10.3390/ma19071455

**Published:** 2026-04-05

**Authors:** Mateusz Mientki, Anna Zawadzka, Magdalena Kowalska, Michał Zawadzki, Amal Tarbi, Bouchta Sahraoui, Przemysław Płóciennik

**Affiliations:** 1 Institute of Physics, Faculty of Physics, Astronomy and Informatics, Nicolaus Copernicus University in Torun, Grudziadzka 5, 87-100 Torun, Poland; 2 Centre for Modern Interdisciplinary Technologies, Nicolaus Copernicus University, Wilenska 4, 87-100 Torun, Poland; 3 Department of Mechanical Engineering, Politecnico di Milano, Via Giuseppe La Masa 1, 20156 Milan, Italy; 4 Scientific Research and Innovation Laboratory, Superior School of Technology, Ibn Tofail University, P.O. Box 242, Kenitra 14000, Morocco; 5 SFR MATRIX, LPhiA, University of Angers, Bd Lavoisier 2, CEDEX 2, 49045 Angers, France; 6 Institute of Engineering and Technology, Faculty of Physics, Astronomy, and Informatics, Nicolaus Copernicus University in Torun, Wilenska 7, 87-100 Torun, Poland

**Keywords:** transparent conductive oxides, copper-doped zinc oxide layers, physical vapor co-deposition, annealing, transmission, energy bandgap, voltage-current characteristics, atomic force microscopy, X-ray fluorescence, thin-film perovskite solar cells

## Abstract

**Highlights:**

- The vapor phase co-deposition technique was successfully implemented for ZnO:Cu thin films.
- An annealing process was performed to modify the optical and electrical properties.
- AFM imaging and qualitative and quantitative XRF composition analysis were performed.
- ZnO:Cu layers exhibit high transparency, uniformity and ohmic conductivity over a wide temperature range.
- A complete structure for a thin-film perovskite solar cell with an efficiency of 17% was proposed.

**Abstract:**

Indium Tin Oxide (ITO) is one of the most widely used ohmic materials for fabricating ohmic layers in thin-film solar cells. ITO thin layers have reached almost the maximum theoretical conductivity and the lowest practical resistivity. Along with indium’s toxic environmental impact and the high cost of materials, these are the reasons why new materials for efficient, cheaper thin-film transparent ohmic layers are being examined. One of those materials is copper-doped zinc oxide (ZnO:Cu). In this paper, we present a new approach to copper-doped zinc oxide fabrication methods, based on the modern authorial Physical Vapor Co-Deposition technique, which involves optimizing Cu concentration to fine-tune crystal structure, optical band gap, and electrical properties, creating n-type TCOs essential for efficient charge transport in next-generation thin films perovskite solar cells.

## 1. Introduction

In recent years, thin-film transparent photovoltaic (PV) cells have attracted increasing interest in the field of renewable energy sources. Their lower material consumption and mechanical flexibility, compared to those of the most commonly used silicon PV cells, provide a significant advantage in developing and fabricating modern solar cells [1,2]. Some projects assume that, in the future, it will be possible to use thin-film PV cells by depositing them on curved and flexible surfaces, e.g., cars and clothing [3,4].

The key component of a thin-film PV cell is the ohmic layer, which serves as the front electrode. Common materials used to manufacture ohmic layers are transparent conductive oxides (TCOs). As the name suggests, their properties are high conductivity and transparency in the visible range [5]. The industry-standard material with excellent optoelectronic properties mentioned above is indium tin oxide (ITO), which is widely employed as an ohmic contact layer in thin-film solar cells, displays, and optoelectronic devices [6,7]. Despite its advantages, ITO exhibits significant drawbacks. Indium is a scarce and expensive element, raising concerns about long-term supply security and cost scalability [6,8]. ITOs’ limitations have motivated extensive research into alternative TCO materials based on earth-abundant and non-toxic elements.

Transparent conductive copper-doped zinc oxide (ZnO:Cu) films are developed for perovskite solar cells (PSCs) to replace costly Indium Tin Oxide (ITO) as electrodes, offering better performance, especially on flexible substrates, using techniques like sol–gel or sputtering, by enhancing conductivity and stability through Cu doping, leading to lower resistivity, high transparency, and improved PSC efficiency. In this context, zinc oxide (ZnO) has emerged as a promising TCO host material due to its wide band gap, high transparency, chemical stability, and compatibility with various deposition techniques [5,9]. Recent studies show that doping ZnO with various metals, such as aluminum, enhances its conductivity, and some of them indicate that copper may also offer favorable electrical properties while maintaining high optical transparency [10,11]. Owing to the abundance and low cost of zinc and copper, ZnO:Cu represents a potential next-generation TCO material suitable for thin-film photovoltaic applications, particularly where flexibility, sustainability, and scalability are critical.

The further parts of this paper explain the process of manufacturing thin film ZnO:Cu samples in Section 2 using the physical vapor deposition method and annealing. In Section 3, the samples’ opto-electrical properties are measured using UV-Vis and XRF spectroscopies and I-U measurements, and their topography is characterized using Atomic Force Microscopy (AFM) and X-Ray Fluorescence Spectroscopy (XRF). The final results compare the best results from the work with data from other papers, andthe obtained results are used to propose a structure for a thin-film perovskite solar cell.

## 2. Materials and Methods

### 2.1. Physical Vapor Deposition

**Figure 1:**
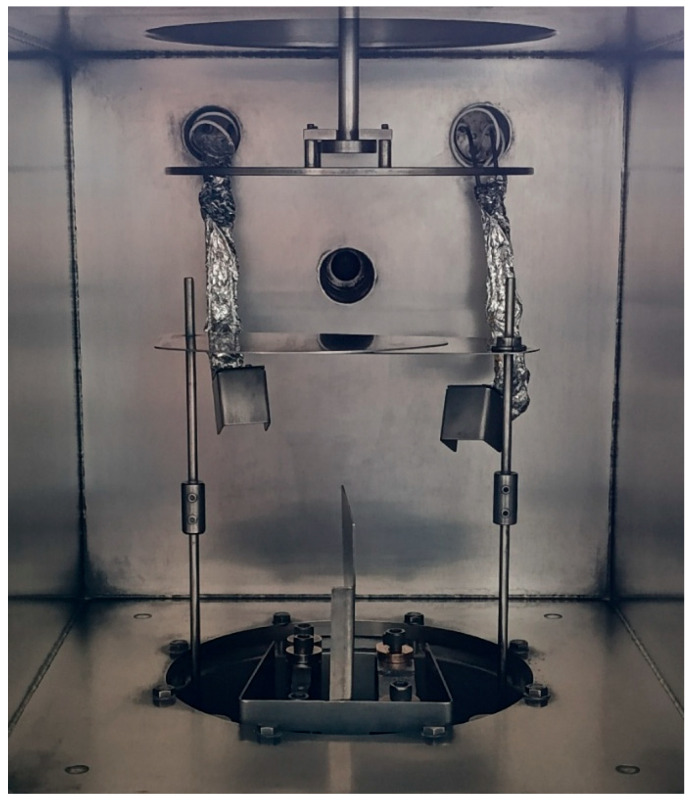
The inside of a vacuum chamber used in the Thin Film Deposition System—NANO 36™ (PVcoD process).

The ZnO:Cu samples were obtained using the Physical Vapor Co-Deposition (PVD) method. The apparatus used for manufacturing was the Thin-Film Deposition System—NANO 36™ (Kurt J. Lesker Company (Jefferson Hills, PA, USA)—Figure 1) [12,13,14]. The material in the pure form of dust (Zn) or a chunk of metal (Cu) was placed on the heating material at the bottom of the vacuum chamber. During the deposition process, the substrate (glass) was placed on the rotating panel at the top of the chamber at room temperature and rotated at a speed of 20 rot/min. The deposition occurred under the pressure of approximately 2–3 × 10^−6^ Tr, which reduced atmospheric contamination, extended the free path of the material, and lowered the temperature required for material evaporation. The long distance between the source of material and the substrate (~20 cm), along with the rotational panel, was implemented to minimize the thickness difference across the sample surfaces due to scattering. The zinc and copper were heated separately, due to differences in their evaporation temperatures, by applying specific voltage and current values to the source’s heating components, ensuring precise doping levels. The evaporation speed of each material was monitored using piezoelectric sensors. Once the optimal evaporation speed was determined, the shutters between the source and substrate were opened, initiating the deposition process. The Cu doping levels in the ZnO layers obtained in the experiment were 0% (pure ZnO), 2.5%, 5%, and10%.

### 2.2. Annealing

**Figure 2:**
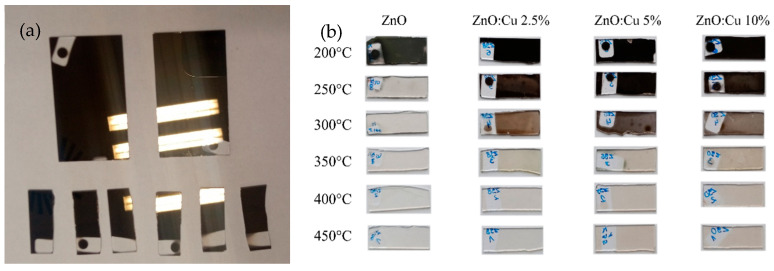
The appearance of samples, (**a**) before annealing and (**b**) after annealing, depending on the level of doping and annealing temperature.

Due to the low oxygen content in the obtained ZnO:Cu samples, their transmission was low, and the samples were highly reflective and dark. To enhance the optical properties and oxygen amount in the samples, they were annealed at temperatures from 200 °C to 450 °C. The difference in appearance of the samples before and after annealing at the given temperature is shown in Figure 2.

## 3. Results and Discussion

In this chapter, the measurements of optoelectrical and topological properties are presented. The most optimal results were obtained for the 5% Cu-doped ZnO sample annealed at a temperature of 300 °C; thus, the results of measurements are mainly discussed based on this sample.

### 3.1. Transmission Measurements

**Figure 3:**
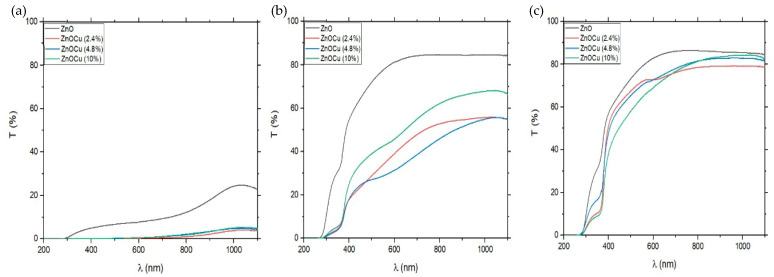
The transmission levels of ZnO:Cu samples on the glass substrate (**a**) before annealing, (**b**) after annealing at 300 °C, and (**c**) after annealing at 350 °C.

**Figure 4:**
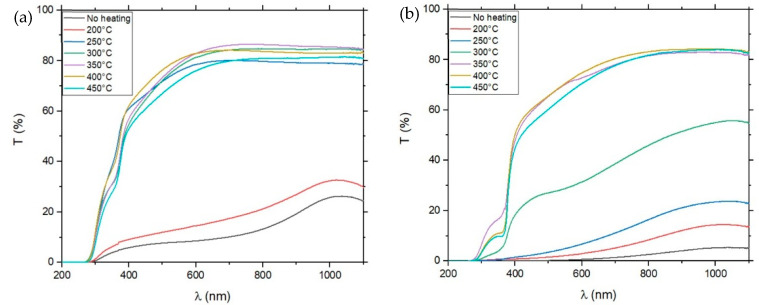
Transmission levels of the sample on the glass substrate, pure ZnO (**a**) and 5% Cu-doped ZnO (**b**), depending on annealing temperature.

Annealing of the samples resulted in a significant increase in sample transmission. Figure 3 shows the transmission levels of samples before and after their annealing under temperatures of 300 °C and 350 °C, and Figure 4 shows the results of transmission measurements of pure ZnO and 5% Cu-doped ZnO annealed in temperatures from 200 °C to 450 °C. The transmission level slightly increased after annealing the samples at 200 °C and 250 °C. The significant rise occurred after annealing at 300 °C and 350 °C. Heating samples at higher temperatures (400 °C and450 °C) did not have a greater influence on transmission results. Cu doping loweredtransmission at 300 °C and below; at higher temperatures, the doping did not significantly affect transmission. The percentage of Cu-doping in samples did not have a significant effect on the transmission measurements—all doped samples remained at a similar level in terms of transmission for the whole spectrum, especially at higher annealing temperatures.

### 3.2. Energy Bandgaps

**Figure 5:**
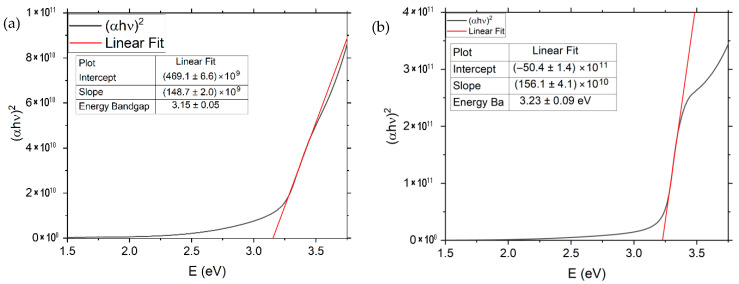
Taucplots and extrapolation of linear fragments leading to the identification of the energy bandgap of (**a**) pure ZnO and (**b**) ZnO:Cu5% samples.

Transmission spectra of ZnO and Cu-doped ZnO thin films were analyzed to determine the optical band gap using the Tauc method [15,16]. The absorption coefficient *α* was calculated from the transmittance *T* according to the following:

(1) $$\alpha =\frac{1}{d}\ln\left(\frac{1}{T}\right)$$

where *d* = 150 nm is the film thickness. Assuming allowed direct transitions, the quantity (*αhν*)^2^ was constructedas follows:

(2) $${\left(\alpha h\nu \right)}^{2}={\left(\frac{h\nu }{d}\ln\left(\frac{1}{T}\right)\right)}^{2}$$

where *h* is Planck’s constant and *ν* is the photon frequency. The linear part of the (*αhν*)^2^ vs. photon energy *E* plot was extrapolated to zero to obtain the optical band gap, reflecting the onset of interband electronic transitions. Figure 5 shows the difference in Tauc plots and linear fits between pure ZnO and ZnO:Cu. Table 1 presents the specific results of energy bandgaps obtained for each copper concentration in ZnO samples and compares them with results from other scientific works [17,18,19,20,21]. The comparison indicates that Cu doping of ZnO increases the energy bandgap, contrary to the results reported in other scientific works. Conversely, the findings from alternative methods [22,23] demonstrate the potential for enhancing the energy bandgap through various physical phenomena, such as the Burstein–Moss effect, which exerts a more substantial influence on outcomes when compared to impurity band formation or p–d coupling. This finding indicates that doping enhances the concentration of free carriers and impurity states in the conduction band, as opposed to within the bandgap. Consequently, the Fermi level increases and the optical bandgap widens rather than narrowing.

### 3.3. I(U)Characteristics and Sheet Resistance of ZnO:Cu

**Figure 6:**
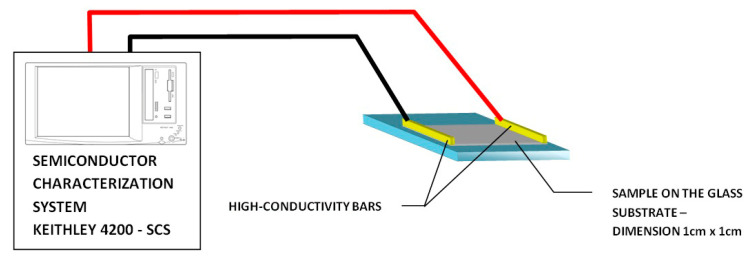
Diagram of the measurement system for testing the electrical properties of the ZnO:Cu layers (Keithley Instruments, Tektronix company, Cincinnati, OH, USA).

The sheet resistance of ZnO:Cu before annealing ranged from 200 to 400 Ω/sq. After annealing, the *I*(*U*)characteristics of the samples were obtained using a Keithley Model 4200-SCS Semiconductor Characterization System at various temperatures from 10 K to 300 K, controlled with a Janis Cryogenics Model 22 cryostat and a Lake Shore 335 temperature controller (Janis Research, Wilmington, MA, USA) [24,25,26]. The initial test measurements were performed using a four-wire method and compared with the results obtained using high-conductivity bar electrodes placed on opposite edges of the sample, as shown in Figure 6, yielding consistent results. All surface resistance measurements as a function of temperature were performed similarly.

**Figure 7:**
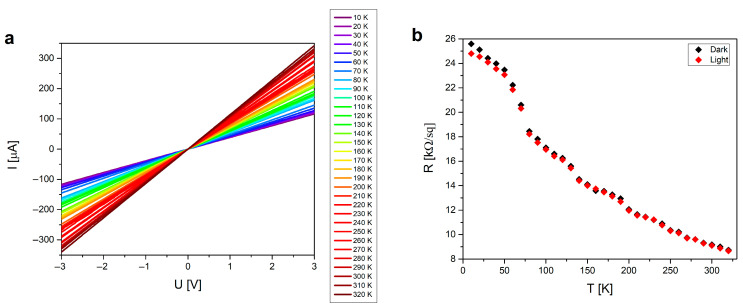
(**a**) The I-U characteristics of 5% Cu-doped ZnO samples annealed at 300 °C depending on the temperature of annealing, and (**b**) the sheet resistance values of samples depending on the temperature of annealing, before and after exposing the samples to light for 1 min.

The I-U characteristics of ZnO doped with 5% Cu, annealed at 300 °C, are shown in Figure 7a, and it shows that the sheet resistance of samples increased by twoorders of magnitude due to annealing; with ambient temperature, the current increased as the sheet resistance decreased (Figure 7b). Furthermore, Figure 7b presents that shining light on samples during I-U measurements works asa benefit, further lowering the sheet resistance of samples.

**Figure 8:**
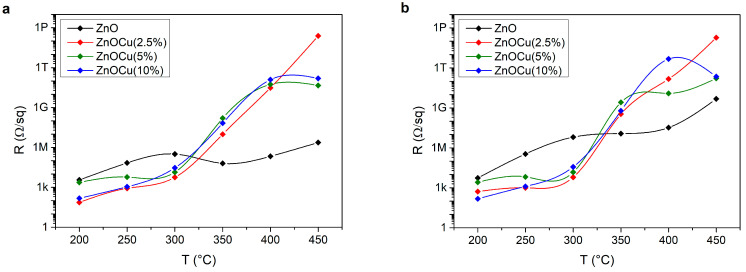
Sheet resistance of ZnO:Cu samples depending on the temperature of annealing and intensity of doping (**a**) before (**b**) and after the illumination of the samples.

Further analysis of ZnO:Cu samples shows that annealing the samples at temperatures above 300 °Ccausedthem to overheat and reachenormous values of sheet resistance, in the order of 1 TΩ and higher (Figure 8). It also shows that illuminating the samples allowedfor a reduction in sheet resistance for some high-resistance samples.

For transparent conductive electrodes, sheet resistance, along with optical transmittance, is typically used to calculate the Figure of Merit (FoM), which can be used for comparison with other transparent electrode materials, e.g., ITO. The Figure of Merit (FOM Haacke High Resolution) was calculated from the relationship proposed by Cisneros-Contreras et al. [27].

(3) $${FOM}_{H-HR}=\frac{T}{\sqrt[{n}]{R_{S}}}$$

where *T* is the transmittance, *R_S_*—sheet resistance and n = 10. Calculated value of FOM is presented in Table 2.

### 3.4. Atomic Force Microscopy

**Figure 9:**
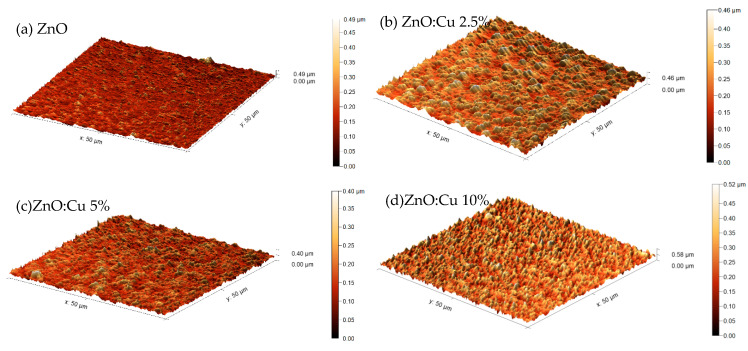
The 3D visuals of the surfaces of manufactured samples. (**a**) Pure ZnO, (**b**) ZnO:Cu 2.5%, (**c**) ZnO:Cu 5%, and (**d**) ZnO:Cu10%.

Atomic Force Microscopy (AFM) measurements were taken in contact mode using a Sicon-A tip to characterize the surface quality of manufactured samples. The result of the AFM measurement is shown in Figure 9. The Cu-doping in ZnO layers causes more grains and irregularities to appear in the structure. For pure ZnO, the average surface height, compared to the lowest value measured, is 150.1 nm; for ZnO:Cu 5%, it is slightly higher: 153.6 nm; for the remaining samples, it is the highest, at 229.1 nm for ZnO:Cu 2.5% and 247.7 nm for ZnO:Cu 10%.

**Figure 10:**
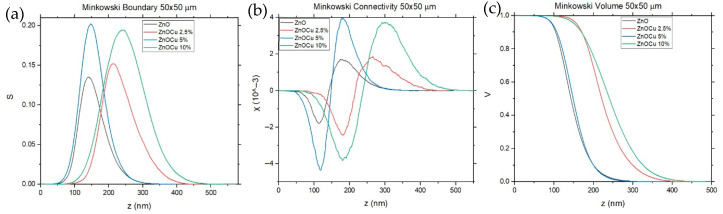
Minkowski diagrams: (**a**) Boundary, (**b**) Connectivity, and (**c**) Volume.

For further analysis of AFM images, the Minkowski Functionals are characterized. Volume, Boundary, and Connectivity (presented in Figure 10) fully characterize the surface morphology in terms of height distribution, interfacial complexity, and topological arrangement of surface features.

The comparison between undoped ZnO and ZnO:Cu 5% reveals that moderate Cu incorporation preserves the overall morphological characteristics of the ZnO surface while inducing subtle but significant structural refinement. The Minkowski Volume curves for both samples exhibit similar shapes and threshold positions, indicating comparable global height distributions and average surface roughness. This similarity suggests that 5% Cu doping does not fundamentally alter the vertical growth mode of ZnO. Apparent differences emerge in the Minkowski Boundary and Connectivity functionals. ZnO:Cu 5% exhibits a noticeably higher Boundary maximum than undoped ZnO, reflecting an increased total interfacial length between regions of different height. This behavior indicates finer lateral grain segmentation and a higher grain-boundary density. At the same time, the Connectivity remains predominantly positive across a broad threshold range, indicating a surface composed of well-separated, topologically stable surface features with limited lateral percolation. Compared to that of ZnO, the ZnO:Cu 5% surface therefore combines preserved height uniformity with enhanced geometric complexity and controlled topological order. Such a morphology is advantageous for thin-film quality, as it implies controlled grain refinement without the formation of extended porous networks. The increased boundary density may enhance defect accommodation and strain relaxation, while the absence of excessive Connectivity suggests stable surface continuity, both of which are beneficial for electrical and optical performance [9,10,28].

In contrast, both lower (2.5%) and higher (10%) Cu doping levels result in surface morphologies that deviate significantly from that of the ZnO reference. In both cases, the Minkowski Volume curves shift toward higher height thresholds and exhibit broader decays, indicating increased vertical inhomogeneity and pronounced surface irregularities. The corresponding Boundary functionals exhibit broadened, shifted maxima, reflecting increased geometric disorder and a more irregular grain structure. Furthermore, the Connectivity functionals for both doping levels display extended regions of a reduced or negative Euler characteristic, indicating enhanced lateral interconnection and the development of percolating or porous surface networks. These observations suggest that both insufficient and excessive Cu incorporation destabilize the ZnO growth process, promoting morphological irregularities and topological disorder rather than controlled surface refinement [29,30,31].

### 3.5. X-Ray Fluorescence

X-ray fluorescence spectroscopy (XRF) is a widely used, non-invasive analytical technique for identifying and quantifying elements in materials of various physical forms, including solids, powders, and liquids [14,32,33]. This method is based on the emission of characteristic X-ray radiation by atoms in a sample upon excitation by an external X-ray source. During measurement, the sample is irradiated with X-rays; electrons are ejected from inner atomic shells, and electrons from higher-energy shells migrate to fill the gaps, emitting radiation with energies characteristic of the individual elements. Analysis of the energy and intensity of the emitted radiation enables both the qualitative identification and quantitative determination of elements with minimal sample preparation and without sample destruction. A micro-X-ray fluorescence spectrograph (μXRF) from Bruker Nano GmbH was used for quantitative and qualitative analysis of the composition of the studied thin transparent conductive layers. This spectroscopy provides advanced analytical capabilities for elemental analysis across research and industrial applications. Its key components include tunable X-ray sources, highly sensitive detectors with excellent energy resolution, and precise sample-positioning mechanisms that enable elemental mapping at the thin-film surface. This μXRF spectrograph allows for the mapping of elemental distributions over large areas while maintaining high spatial resolution, facilitating the analysis of micro-regions and the detection of chemical compositional inhomogeneities within thin-film structures. Bruker ESPRIT (XRF/SEM software provides miscellaneous control over data acquisition and spectra analysis. This software enables the acquisition of both qualitative and quantitative XRF spectra, the identification of characteristic elemental lines, and the generation of elemental distribution maps [34].

In practice, XRF allows the determination of most elements with atomic numbers typically above 11–13. However, the detection limits and analytical accuracy depend on the type of instrumentation, measurement geometry, and the physical and chemical properties of the sample. This means that elements such as hydrogen, nitrogen, carbon, and oxygen are not visible in the X-ray fluorescence spectra of the materials. Therefore, further quantitative and qualitative analyses of the investigated transparent conductive layers are limited to comparisons of the characteristic XRF lines for atoms of zinc and copper.

**Figure 11:**
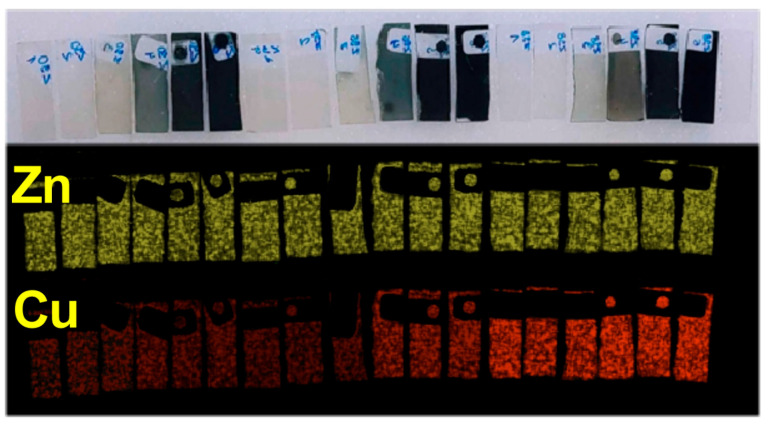
XRF images—analysis of elements in the ZnO:Cu layer as a function of their percentage content: zinc (dark yellow) and copper (red).

Figure 11 presents the qualitative and quantitative XRF analysis of zinc and copper in the investigated thin, transparent ZnO:Cu layers. X-ray fluorescence imaging yields elemental distribution maps that represent the spatial distribution of chemical elements across the sample surface. Each pixel of an XRF map corresponds to a full fluorescence spectrum acquired at a specific position on the sample. This approach enables the determination of the spatial distribution and relative concentrations of individual elements, as well as the assessment of the compositional homogeneity of the ZnO matrix and the uniformity of copper doping.

Qualitative evaluation of the XRF maps (Figure 11) indicates a high degree of chemical homogeneity over the entire analyzed surface of the transparent conductive layers. Both zinc and copper are uniformly distributed across the sample area. Minor local variations in signal intensity observed between individual pixels are attributed primarily to the surface roughness of the films, which is consistent with the morphological features revealed by AFM measurements. The quantitative information, expressed in the maps as variations in fluorescence intensity, is particularly pronounced for the copper dopant. A systematic decrease in the Cu K-line fluorescence intensity is observed as copper concentration decreases in the investigated layers.

**Figure 12:**
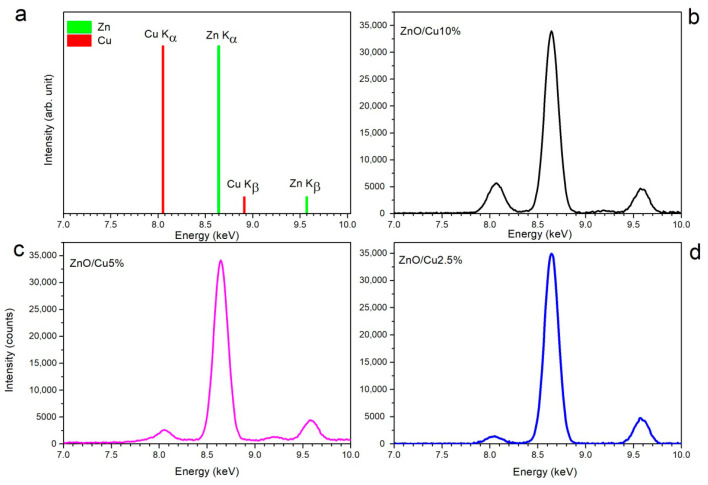
XRF spectra: characteristic emission lines of zinc and copper (**a**), XRF spectrum of a ZnO:10%Cu sample (**b**), XRF spectrum of a ZnO:5%Cu sample (**c**) and XRF spectrum of a ZnO:2.5%Cu sample (**d**).

Detailed quantitative analysis was performed by comparing the elemental composition within selected regions of interest of the samples. For each layer, an area of 0.5 cm^2^ (0.5 cm × 1 cm) was analyzed to ensure representative sampling. The resulting spectra provided information on the energies of the characteristic fluorescence lines and their corresponding intensities. Representative XRF spectra acquired from transparent conductive ZnO:Cu layers with different copper contents, annealed at 300 °C, are shown in Figure 12.

The spectra were obtained to identify characteristic emission lines of zinc and copper and to assess the relative changes in elemental content as a function of dopant concentration. These spectra were obtained after subtracting the XRF spectra obtained for transparent coatings on a glass substrate and a clean glass substrate. This approach allowed for easy analysis of the characteristic lines in the XRF spectrum. Figure 12a shows the reference Zn and Cu spectra used for peak identification. Characteristic emission lines of copper, Cu K_α_ at 8.05 keV and Cu K_β_ at 8.91 keV, are indicated, as are characteristic lines of zinc, with Zn K_α_ at approximately 8.63 keV and Zn K_β_ at 9.57 keV. These reference spectra provide the basis for assigning the fluorescence peaks observed in doped ZnO samples. Figure 12b shows the XRF spectrum of a ZnO:Cu sample containing 10% Cu. Prominent fluorescence peaks corresponding to both the Zn K_α_ and Cu K_α_ lines, as well as the Zn K_β_ line, are clearly visible. The Cu K_β_ line is practically invisible in the spectra, regardless of dopant concentration or annealing temperature. The ratio of the peak intensities for Zn and Cu reflects the percentage of these elements in the studied layer. The intensity of the Zn peaks is approximately 10 times greater than that of the Cu peak. The relatively high intensity of the Cu peak indicates a higher copper content compared to that of samples with lower dopant concentrations. Figure 12c shows the XRF spectrum of the ZnO:Cu sample with 5% Cu. In this case, the Zn K_α_ line is dominant, while the Cu K_α_ line is noticeably weaker than in the sample with 10% Cu. This reduction in the Cu signal reflects the reduced copper concentration in the layer. Figure 12d shows the XRF spectrum of the ZnO:Cu sample with 2.5% Cu. The Zn K_α_ emission dominates the spectrum, while the Cu K_α_ line is barely perceptible. The progressive decrease in the Cu K_α_ peak intensity with decreasing dopant concentration shows a clear and systematic trend, confirming the effective control of copper incorporation into the ZnO matrix.

**Figure 13:**
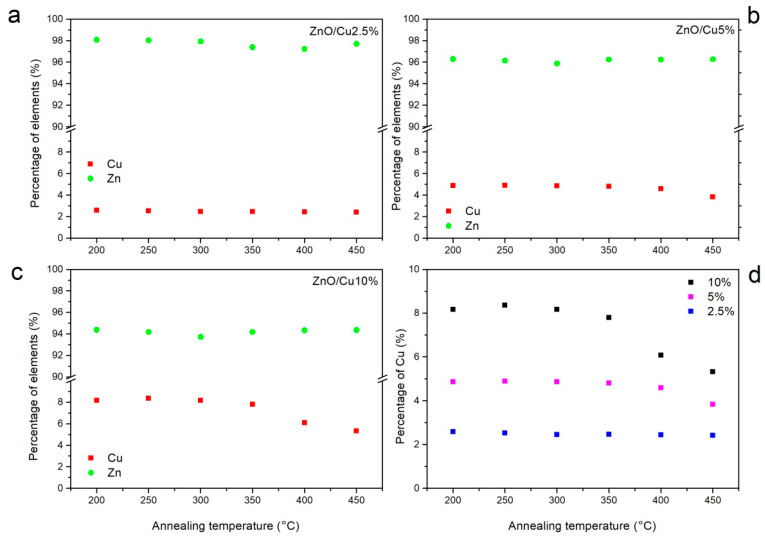
Percentage composition of the main elements depending on the annealing temperature estimated fromthe XRF spectra of the ZnO:10%Cu sample (**c**), ZnO:5%Cu sample (**b**), ZnO:2.5%Cu sample (**a**); percentage of Cu admixture (**d**).

To examine in detail the changes in the intensities of characteristic lines in the emission spectra, a thorough analysis of the XRF spectra was performed as a function of dopant percentage and annealing temperature. The obtained results are presented in Figure 13.

To investigate in detail the variations in the intensities of the characteristic emission lines, a systematic analysis of the XRF spectra was carried out as a function of dopant concentration and annealing temperature. The corresponding results are presented in Figure 13. The determined zinc content remains consistent with the nominal composition of the films with high accuracy. Within the investigated annealing temperature range of 200–450 °C, only minor variations in the Zn content (<1%) were observed. Such behavior is typical for thin ZnO films prepared using both vacuum-based deposition techniques and solution-derived precursor methods. These results indicate that, irrespective of the introduced dopant concentration, the overall stoichiometry and integrity of the ZnO matrix remain essentially unchanged.

In contrast, the copper dopant exhibits markedly different behavior. In the temperature range between 200 and 300 °C, only small fluctuations in the Cu content (<1%) are observed. However, starting from an annealing temperature of 350 °C, a systematic decrease in the copper concentration in the ZnO layers is detected for all investigated doping levels. The magnitude of this decrease becomes more pronounced with increasing nominal Cu content. For the sample containing 10% Cu, the reduction in copper concentration reaches approximately 30% after annealing at 450 °C.

This effect can be attributed to the thermally induced resublimation and outward diffusion of copper from the ZnO layer during the annealing process. Because Cu atoms may occupy both substitutional (Zn sites) and interstitial positions within the ZnO crystal lattice, they exhibit relatively high mobility with increasing temperature. While a fraction of the dopant atoms rearranges into energetically more favorable lattice positions, the remaining copper atoms are expelled from the film, predominantly through the surface. As a consequence, higher annealing temperatures lead to an increasing dopant concentration gradient across the film thickness, with the lowest copper concentration occurring at the surface region.

The depletion of copper at the surface results in a significant increase in the surface resistance of the transparent conductive coating. This behavior is consistent with the electrical measurements performed as a function of annealing temperature (Figure 8), which reveal an increase in sheet resistance by more than four orders of magnitude, from several hundred ohms (Ω/sq) to several hundred teraohms (TΩ/sq) per square.

### 3.6. Thin-Film Perovskite Solar Cell Using ZnO:Cu as a Transparent Conductive Layer

**Figure 14:**
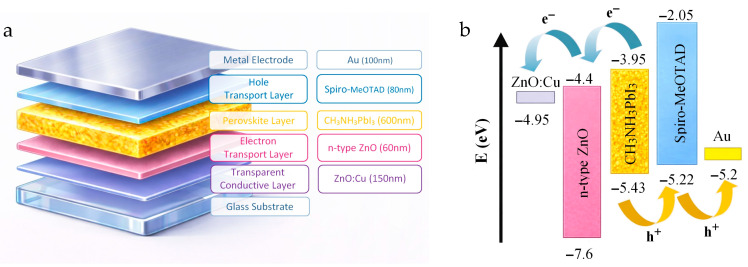
The structure of a thin-film perovskite solar cell (**a**) and thin-film PSC energy diagram (**b**).

The structure of a thin-film perovskite solar cell, shown in Figure 14a, consists of a glass substrate, a transparent conductive layer (TCL), an electron transport layer (ETL), an active perovskite layer, a hole transport layer (HTM), and a metal electrode. Figure 14b shows the thin-film PSC energy diagram.

**Figure 15:**
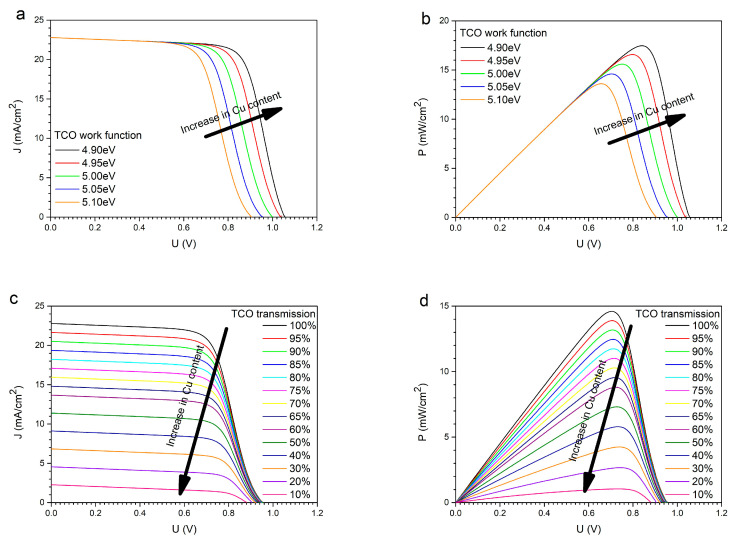
The influence of copper doping of transparent ZnO:Cu conductive oxide layers on the basic characteristics of a thin-film perovskite solar cell: J(U) for different work functions (**a**), P(U) for different work functions (**b**), J(U) for different transmissions of the TCL (**c**) and P(U) for different transmissions of the TCL (**d**).

To demonstrate the influence of the properties of the transparent conductive layer made of copper-doped zinc oxide (ZnO:Cu), simulations of basic cell characteristics were performed, including the dependence of the current density (J) and the power generated by the cell on voltage (U). The J(U) characteristic shows how the output current density J changes as a function of the voltage U at the cell terminals under constant illumination and temperature. The most critical points of this characteristic are the short-circuit current (I_SC_) and the open-circuit voltage (U_OC_). Short-circuit current is the current when the voltage at the cell’s terminals is equal to zero. Physically, it represents the maximum current that the cell can deliver under a given irradiance. Open-circuit voltage is the voltage when the output current is zero. It represents the maximum voltage that appears at the terminals of an unloaded perovskite cell. In turn, the dependence of the power generated by the cell as a function of voltage allows us to determine the cell’s operating area, fill factor (FF), and maximum power point (MPP). The simulations were conducted using our own experimental data for individual layers and SCAPS-1D software, version 3310 ([35,36]. The transparent conductive oxide layer was a 150 nmthick copper-doped zinc oxide layer (ZnO:Cu). The remaining thin cell layers were as follows: 60 nmthick ETL (n-type ZnO), a 600 nmthick perovskite active layer (CH_3_NH_3_PbI_3_), an 80 nmthick HTL (SpiroMeOTAD), and a 100 nmthick gold (Au) metallic electrode [12,37,38]. In the simulations, only the transparent conductive oxide (TCO) parameters were changed; the remaining layer parameters in the thin-film perovskite solar cell remained unchanged. Figure 15 shows the behavior of the basic characteristics of a thin-film perovskite solar cell depending on the Cu content of the TCLs.

**Figure 16:**
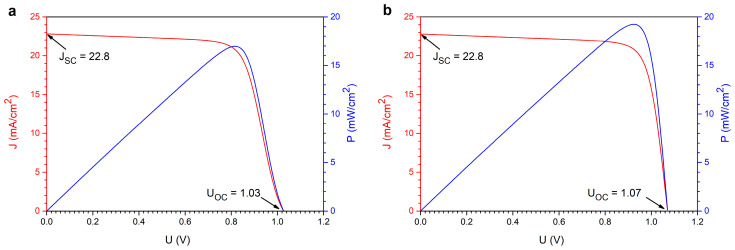
J(U) and P(U) characteristics for the optimized thin-film perovskite cell structure with different TCO layers: ZnO:Cu (**a**) and ITO (**b**).

Copper doping of ZnO layers primarily shifts the Fermi level of the resulting TCL. The average work function of pure ZnO is 5.13 eV, while that of Cu is 4.7 eV, which is lower than that of ZnO. Therefore, band bending is observed, leading to the formation of an electron accumulation region. This region forms at the Cu/ZnO interface, affecting the work function. Since ZnO is an n-type material, a shift in the Fermi level toward the conduction band indicates an enhancement of the n-type character. Copper, therefore, acts as an electron donor, thereby reducing the sample’s resistivity. Thus, Cu-doped ZnO layers can be used as TCOs, with sheet resistances ranging from a few to several hundred ohms per square. From a purely electrical perspective, the more copper in the ZnO structure, the lower the TCL resistance. In practice, lowering the ZnO:Cu work function to the value of pure copper would yield the best conductivity results. This, in turn, increases the cell’s open-circuit voltage, expands its operating range, and improves cell efficiency. On the other hand, copper doping reduces the transparency of the ZnO layers, meaning less sunlight reaches the deeper layers of the cell. This reduces short-circuit current, narrows the operating region, and lowers cell efficiency. Therefore, it is necessary to find an appropriate balance in the Cu doping of ZnO layers for practical use as transparent, conductive oxide layers. The highest cell efficiency is achieved with doping levels below 10%, though this also depends on the placement of the ETL material’s conduction and valence band energy levels. For the studied thin-film perovskite solar cell structure, the highest efficiency was obtained with transparent ZnO:Cu conductive oxide layers containing 5% copper. Figure 16 shows the J(U) and P(U) characteristics for the optimized cell structure with different TCO layers, ZnO:Cu (Figure 16a) and ITO (Figure 16b), while maintaining the other cell parameters. Table 3 includes the estimated basic parameters of the thin-film solar cells.

The achieved cell efficiency is approximately 17%, comparable to that of commercially available silicon cells. The advantage of the proposed cell structure is undoubtedly the use of n-ZnO in its pure form (as the ETL) and doped with Cu (ZnO:Cu; as the TCL). This approach significantly reduces interlayer effects in the cell structure and allows for the free flow of charges from the electron transport layer to the transparent contact layer of the solar cell.

One of the key parameters for assessing energy conversion in solar cells is the external quantum efficiency (EQE). It describes how efficiently incident photons of a given wavelength are converted into collected charge carriers. Analyzing EQE as a function of wavelength can determine which regions of the solar spectrum are effectively utilized and which contribute less to the generated photocurrent. External quantum efficiency at a given wavelength can be defined as follows:

(4) $$EQE(\lambda )=\frac{hc}{e\lambda }\cdot \frac{I_{ph}(\lambda )}{P_{in}(\lambda )}$$

where $I_{ph}(\lambda )$ is the photocurrent density (A/cm^2^)—the current generated by the cell for a given wavelength; $P_{in}(\lambda )$—the power of incident radiation at a given wavelength; *e*—the elementary charge; *h*—Planck’s constant; *c*—the speed of light; and *λ*—a given wavelength.

**Figure 17:**
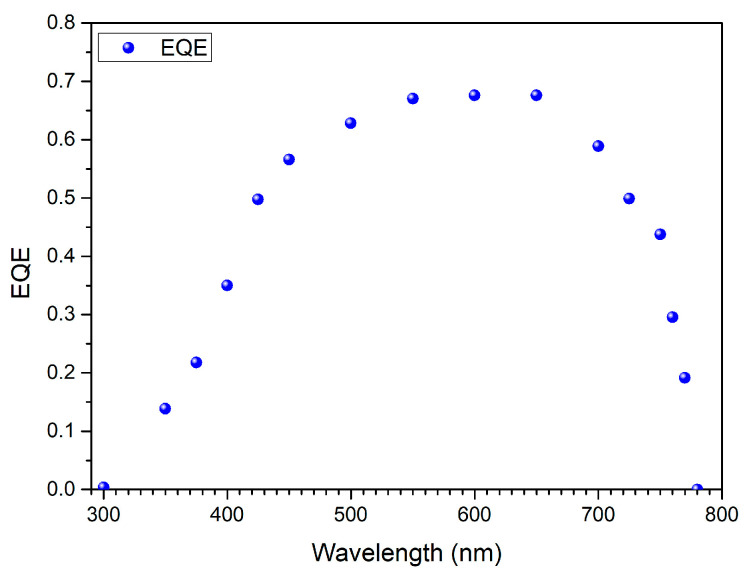
EQE for thin-film PSC using ZnO:Cu as TCL.

The importance of EQE lies in its ability to provide spectrally resolved information on solar cell performance that cannot be obtained solely from overall efficiency measurements. This makes EQE an indispensable tool for identifying spectral losses and optimizing device materials and structures to improve their efficiency. Figure 17 shows that the effective energy conversion range of the proposed cell structure is 300–780 nm. The maximum EQE is 0.7, indicating good energy conversion efficiency.

## 4. Conclusions

This article presents a systematic study of ZnO:Cu thin films intended for use as transparent conductive coatings in thin-film perovskite solar cells. All layers were fabricated using the PVco-D physical co-deposition technique with three copper doping levels (2.5%, 5%, and 10%), and their structural, optical, and electrical properties were correlated with the operating parameters of the final photovoltaic structures. After fabrication, the layers were annealed over a wide temperature range, from 200 to 450 °C, to reduce structural defects associated with oxygen vacancies and increase the transparency of the TCO layers. It was demonstrated that the introduction of Cu ions into the ZnO thin-film structure directly affects the sheet resistance of the TCO layers, which can significantly change the charge-carrier concentration. For 2.5% doping, only a moderate increase in conductivity was observed, resulting in relatively high sheet resistance while maintaining excellent optical transmittance in the visible range. However, for 10% doped layers, a significant deterioration in structural properties was observed, manifested by increased crystal lattice disorder and the probable formation of local Cu clusters or secondary phases, leading to enhanced charge scattering mechanisms and surface layer depletion during the annealing process. The best performance parameters were obtained for 5% doped ZnO:Cu layers. At this concentration, a simultaneous increase in carrier concentration and relatively high carrier mobility is predicted, which may result in minimal sheet resistance and a slight decrease in optical transmittance. Analysis of the transmission spectra confirmed that the 5% doping level maintains high transparency in the 400–1100 nm range, which is crucial for absorption in the active layer of a solar cell. Direct correlation of the layer properties with photoelectric parameters suggests that using ZnO:Cu (5%) as a transparent electrode can lead to an increase in the short-circuit current and fill factor, indicating a reduction in resistive losses at the electrode–active layer interface. At the same time, no significant reduction in open-circuit voltage was observed, indicating that doping does not adversely affect charge separation and transport in the cell structure. The results of the TCO layer parameter tests and theoretical simulations of the cell structure indicate that a 5% Cu doping level is the optimal technological solution, providing the most favorable compromise between the optical and electrical properties of ZnO:Cu layers. Therefore, these layers can be considered a promising alternative to classic, transparent conductive coatings (e.g., ITO) in photovoltaic applications.

## Figures and Tables

**Table 1:** Taucplot results of energy bandgaps obtained experimentally compared with data from other scientific works [17,18,19,20,21].

Experimental Data		Other Scientific Works—Similar Methods		Other Scientific Works—Different Methods	
Cu-Doping [%]	Energy Bandgap [eV]	Cu-Doping [%]	Energy Bandgap [eV]	Cu-Doping [%]	Energy Bandgap [eV]
0	3.15 ± 0.05	0 [17,18,19,20]	3.2–3.5	0 [22,23]	3.09, 3.37
-	-	1 [20]	3.41	1.5 [23]	3.35
2.5	3.23 ± 0.07	2 [21]	3.18	2 [23]	3.50
5	3.22 ± 0.09	6 [21]	3.02	3 [23]	3.48
10	3.20 ± 0.06	10 [21]	2.85	5 [23]	3.31
-	-	25 [20]	2.87	10 [22]	3.14

**Table 2:** Figure of Merit (FOM Haacke High Resolution) for ZnO:Cu layers.

Sample	Annealing Temperature [°C]	Sheet Resistance [Ω/sq]	Transmittance at 750 nm	FOM_H-HR_, n = 10 [Ω/sq]^1/10^
ZnO:Cu (2.5%)	200	74.4	0.039	0.025
	250	842.4	0.180	0.092
	300	5701.2	0.566	0.238
	350	9.9 × 10^6^	0.863	0.172
	400	3.1 × 10^10^	0.951	0.085
	450	2.4 × 10^14^	0.943	0.034
ZnO:Cu (5%)	200	2385.6	0.074	0.034
	250	6016.7	0.152	0.064
	300	13,814.3	0.472	0.182
	350	1.6 × 10^8^	0.890	0.135
	400	5.6 × 10^10^	0.912	0.077
	450	4.6 × 10^10^	0.890	0.076
ZnO:Cu (10%)	200	150.3	0.057	0.034
	250	1105.1	0.197	0.098
	300	29,372.3	0.657	0.235
	350	6.8 × 10^7^	0.882	0.145
	400	1.3 × 10^11^	0.914	0.071
	450	1.6 × 10^11^	0.916	0.069

**Table 3:** Solar cells parameters calculated from J(U)curve.

TCO Layer Material	Parameter					
	U_OC_ (V)	J_SC_ (mA/cm^2^)	FF (%)	U_MPP_ (V)	I_MPP_ (mA/cm^2^)	η (%)
ZnO:Cu	1.03	22.80	72.46	0.82	20.80	16.97
ITO	1.07	22.80	78.85	0.92	20.83	19.24

## Data Availability

The data presented in this study are available upon request from thecorresponding authors. The data are not publicly available due to funding requirements.
